# Off-label policy through the lens of trazodone usage and spending in the United States

**DOI:** 10.1093/haschl/qxaf114

**Published:** 2025-06-11

**Authors:** Srikanth Kadiyala, Matthew Chenoweth, Jonathan H Watanabe

**Affiliations:** UCLA Center for Health Policy Research, UCLA Department of Health Policy and Management, UCLA Fielding School of Public Health, Los Angeles, CA 90024, United States; UCLA Department of Health Policy and Management, UCLA Fielding School of Public Health, Los Angeles, CA 90024, United States; UCSF Department of Clinical Pharmacy, UCSF School of Pharmacy, San Francisco, CA 94143, United States

**Keywords:** off-label use, pharmaceutical expenditures, evidence-based medicine, economics

## Abstract

Off-label prescribing—when medications are used for indications not approved by the Food and Drug Administration—is widespread in the US health care system. This study used trazodone, a drug approved in 1981 for depression, as a case study to examine broader issues surrounding off-label utilization and spending. Although only approved to treat depression, trazodone is frequently prescribed off-label for indications of uncertain clinical value (insomnia, anxiety). Using nationally representative data from the Medical Expenditure Panel Survey, we estimated that approximately 24 million trazodone prescriptions were filled in 2019, with health care spending of $294 million. At least 85% of prescriptions (∼20 million) and 84% of spending ($247 million) were for off-label indications, primarily insomnia. Health plan reimbursement per prescription was nearly identical for on-label and off-label use, despite the significant evidence gap. These findings illustrate the scale and inefficiency of off-label prescribing and highlight challenges facing clinicians, patients, and payers. We propose a set of policy solutions—including public and private investment in evidence generation, pricing drugs to account for off-label use, and value-based reimbursement—to advance a more efficient system of off-label use. Trazodone presents a revealing case of the broader systemic problem of off-label prescribing for indications of uncertain clinical value.

## Introduction

The US Food and Drug Administration (FDA) restricts the marketing and promotion of prescription pharmaceuticals to “on-label” therapeutic indications for which products have met rigorous safety and efficacy standards. However, it is common practice for physicians to prescribe FDA-approved drugs for unapproved “off-label” uses based on their clinical judgment and evidence from the scientific literature. Off-label use is widespread. Estimates from a survey of US office-based physicians from 2001 found that 21% of prescriptions written for the pharmaceuticals evaluated were prescribed off-label.^1^ Furthermore, off-label use is initiated rapidly after FDA approval. Berger and colleagues^2^ found that, within 6 months of a new drug's approval by the FDA, off-label use constituted up to 43% of prescriptions. Although comprehensive measurement of off-label drug utilization is sparse, what evidence exists indicates that off-label use varies based on the diseases and treatments that are studied.^1,3,4-7^

Off-label use arises for several conceptual reasons. Off-label use is crucial when FDA-approved treatments are not available. Off-label use for this reason is especially common in special populations such as children and pregnant women, populations which have historically been excluded from randomized controlled trials [RCTs] due to concerns regarding safety. Off-Label use is also important when on-label treatments are available but have proven unsuccessful for the patient. In these situations, clinicians use their professional judgment to determine the appropriateness of use, even when effectiveness and safety information is lacking. Finally, off-label utilization also plays a significant role in patient care with respect to experimentation. In these experimental situations, physicians conjecture health benefits based on observational studies and theory.

However, off-label prescribing may lead to substantial utilization of pharmaceuticals of uncertain or even negative health value and increase health expenditures. History has provided salient examples of the latter. For example, hormone replacement therapy (HRT) was approved by the FDA for treatment of vasomotor symptoms associated with menopause. Subsequently, based on observational data analyses, physicians began prescribing HRT for primary prevention of coronary heart disease.^8,9^ When evaluated properly via an RCT, HRT was shown to increase the incidence of coronary heart disease and breast cancer.^10,11^ In yet another striking example, low-dose aspirin was prescribed off-label for primary prevention of coronary heart disease. The expectation that aspirin would be effective for the primary prevention of cardiovascular disease was theoretically based on its effects on platelet aggregation and blood clot formation. After years of off-label use, the Aspirin in Reducing Events in the Elderly (ASPREE) clinical trial was undertaken to test whether the use of aspirin by older patients was beneficial for primary prevention and disability-free survival.^12^ The ASPREE trial demonstrated that this seemingly harmless form of off-label experimentation increased the risk of major bleeding without a net clinical benefit in this population. Similar harmful effects were identified for off-label use of erythropoietin for anemia associated with critical illness and fenfluramine/phentermine for weight loss with respect to patient outcomes and increased health care expenditures. These examples demonstrate a need for research on untested indications to support policy that will promote safe and effective off-label use.

## Examining off-label use via an analysis of trazodone

This article examines off-label utilization and spending on trazodone, a serotonin receptor antagonist and reuptake inhibitor that is FDA approved for the treatment of patients with major depressive disorder. We analyzed trazodone as a case study to explore broader issues related to off-label drug use and to propose potential policy solutions.

Initially approved by the FDA in 1981 for the treatment of major depressive disorder, trazodone has been increasingly used over time as an off-label treatment for insomnia and anxiety.^13-18^ Prior work studying trazodone usage has been conducted using physician office visits data,^13,14^ National Health and Nutrition Examination Data,^16^ Marketscan claims data,^17,18^ and Medicaid data from West Virginia.^15^ These papers were aimed at analyzing trazodone use in the treatment of insomnia and evaluating time trends in trazodone use in the treatment of insomnia. The clinical and pharmacological rationale for trazodone for insomnia management is predicated on dose-dependent mechanisms of action. This mechanism entails purported sedative hypnotic effects at low doses attributable to antagonism of 5-HT2A receptors, H1 histamine receptors, as well as α_1_-adrenergic receptors.^19^ Compared with FDA-approved insomnia drugs, trazodone is not a controlled substance, which might make trazodone a more appealing choice for physicians and patients.^14^

Although use of trazodone for off-label indications, especially insomnia, is widespread, there is considerable uncertainty regarding its effectiveness. For example, the American Academy of Sleep Medicine's clinical practice guidelines^20^ state that physicians should not use trazodone as a treatment for sleep-onset or sleep-maintenance insomnia. Similarly, the American College of Physicians^21^ concluded that there is insufficient evidence to support the effectiveness of trazodone with respect to sleep outcomes; the 2023 American Geriatrics Society Beers Criteria Update Expert Panel included trazodone as a potentially inappropriate medication in older adults due to concerns related to the risk of falls and confusion.^22,23^ In contrast, 2 systematic reviews^24,25^ concluded that trazodone is an effective treatment for insomnia. Yi et al,^25^ in particular, concluded that trazodone increased perceived sleep quality among patients.

We focus on trazodone as a case study to inform off-label policy for 2 key reasons. First, as noted, existing research has shown that trazodone has been used increasingly off-label for the treatment of insomnia. High-utilization off-label cases are particularly important to examine since any associated benefits, harms, or inefficiencies have broader health implications. Second, while prior studies have examined trazodone utilization, they have not examined spending on trazodone or reimbursement differences between off- and on-label indications. Widespread utilization suggests potentially large aggregate expenditures, yet the exact magnitude of the spending remains unknown. Pharmaceutical spending is a critical metric in the context of off-label use, as it provides a potential scale of the opportunity costs in the health care system, particularly if these expenditures do not yield clinical benefits. Pharmaceutical spending also importantly serves as a benchmark for evaluating the cost of generating evidence on clinical efficacy. Therefore, our analyses of trazodone utilization and spending provide valuable context for policy discussions and solutions aimed at designing a more efficient system for off-label utilization.

## Methods

### Data

We used data from the 2019 Medical Expenditure Panel Survey (MEPS) to analyze trazodone utilization and spending. The MEPS data provides demographic and health care utilization information on a nationally representative sample of noninstitutionalized US individuals. The MEPS data is conducted annually and collects detailed health care utilization and health care reimbursement information from approximately 30 000 respondents to represent the population size and demographics of the United States. We chose to analyze data from 2019 since it predated the COVID-19 pandemic, which had substantial effects on prescription drug utilization.

Although widely used in health policy and health economics research since the late 1990s, the MEPS has been underutilized^26-29^with respect to the study of off-label use, even though it provides direct linkages between prescriptions and the conditions for which individual medications were prescribed. This information is not available in widely used claims databases, and therefore MEPS data uniquely allows for evaluation of on-label vs off-label use and reimbursement at the prescription level.

### Determining on-label vs off-label

To determine whether a trazodone prescription was on-label vs off-label, we used MEPS cross-link files to connect each prescription to 1 or more International Classification of Diseases, Tenth Revision (ICD-10), diagnoses. If a trazodone prescription was linked to more than 1 diagnosis, we took a conservative approach and classified it as on-label if at least 1 of the assigned diagnoses was on-label. Additionally, some prescriptions may not be linked to any diagnosis. In 2019, approximately 9% of trazodone prescriptions were assigned to more than 1 diagnosis and 4.5% of prescriptions were reported without an associated diagnosis.

### Expenditure calculations

In addition to capturing health care utilization information, MEPS also gathers data on payer reimbursement and individual out-of-pocket (OOP) spending. This allowed us to identify payer reimbursement and OOP expenditure data associated with each trazodone prescription. Payer reimbursement was also separated into private, Medicaid, and Medicare reimbursement and reimbursement by all other types of insurers (includes US Department of Veteran Affairs [VA], Indian Health Service, and Tricare). We used health care expenditure information to calculate statistics regarding total trazodone spending, payer spending on trazodone, and OOP spending on trazodone, based on whether the prescription was used for on-label vs off-label purposes. Typically, using MEPS data to study payer drug spending is problematic because MEPS does not contain potential rebates to insurers. But historically, rebates occurred only for branded drugs and for generic drugs reimbursed by Medicaid^30,31^; thus, most of the spending associated with trazodone is not subject to this data issue. We return to this issue in greater detail in our analyses and put a bound on the potential rebate to Medicaid for trazodone.

Finally, in addition to calculating aggregate statistics, we also calculated per-prescription reimbursement differences between on-label and off-label trazodone prescriptions, only for prescriptions that are reimbursed by payers. For these analyses we first calculated a raw unadjusted per-prescription reimbursement difference between on-label and off-label trazodone prescriptions. Subsequently, we calculated a regression-adjusted difference, which accounts for variation across payers and the dosage of medication across on-label and off-label trazodone prescriptions.

The present study is reported according to the Strengthening the Reporting of Observational Studies in Epidemiology (STROBE) guidelines for cross-sectional studies.

## Results

### Off-label and on-label trazodone utilization

We began by categorizing trazodone prescriptions based on diagnosis, specifically focusing on prescriptions associated with a single assigned ICD-10 code (87% of all trazodone prescriptions). Trazodone has only been approved by the FDA to treat depression. All other uses of trazodone are off-label and of uncertain value. Table 1 results indicate that, in 2019, there were a total of 20 705 745 prescriptions issued for trazodone with a single assigned diagnosis. Surprisingly, 73.7% of these trazodone prescriptions were for insomnia and other sleep-related disorders. Supplementary analyses of the MEPS data revealed that trazodone was the most frequently prescribed drug for a diagnosis of insomnia (26% of prescriptions), followed closely by zolpidem (20%), findings that are consistent with prior work.^13,14,16^ After insomnia and sleep-related disorders, 8.4% of trazodone prescriptions were for depression and 5.9% of prescriptions were for anxiety disorders. No other single diagnosis accounted for more than 2% of the single-diagnosis trazodone prescriptions. Taken together, these findings imply that 91.6% of trazodone prescriptions with a single diagnosis were for off-label indications of uncertain value.

**Table 1.** Trazodone prescriptions 2019.

	Off-label	On-label
Single diagnosis prescriptions	18 958 167 [16 282 322–21 634 046]	1 747 578 [831 522–2 663 601]
Multiple diagnoses prescriptions	1 330 246 [682 917–1 977 542]	827 757 [348 448–1 307 066]
Prescriptions with no diagnoses	1 070 456 [414 582–1 726 363]	
Total prescriptions	23 934 204 [20 800 003–27 068 438]	

Source: 2019 Medical Expenditure Panel Survey (MEPS) data. 95% CIs in parentheses. Trazodone has only been approved by the Food and Drug Administration (FDA) for the treatment of depression. All other uses of trazodone are considered off-label.

Next, we turn our attention to off-label and on-label use among trazodone prescriptions with multiple associated diagnoses (9% of all trazodone prescriptions). We characterized a prescription within this group as on-label if at least 1 of the listed diagnoses was for depression and characterized all other claims as off-label. Nearly 62% of this subset of trazodone prescriptions were for off-label indications and the remaining 38% of prescriptions in this group were prescribed for depression alongside at least 1 other diagnosis.

In Table 2, using the definitions of on-label and off-label from above, we examined payer characteristics associated with trazodone prescriptions, since payer characteristics are important with respect to the reimbursement amount. Table 2 shows substantial differences in payer status between on-label and off-label trazodone prescriptions. Interestingly, we found that nearly 32.1% of trazodone prescriptions with diagnoses are wholly self-pay. Of the remaining prescriptions, 15.4% are privately reimbursed, 47.4% are reimbursed by public payors (Medicare and Medicaid), and the remaining 4.8% are reimbursed by other health insurance types.

**Table 2.** Payer composition of off-label vs. on-label trazodone prescriptions with diagnoses.

	Prescriptions	Private payers	Public payers (Medicare and Medicaid)	Other insurance	Uninsured/self-pay
Off-label prescriptions	20 288 413	16.8%	44.7%	5.4%	33.1%
On-label prescriptions	2 575 335	4.3%	71.6%	0.2%	23.9%
Total prescriptions	22 863 748	15.4%	47.7%	4.8%	32.1%

Source: 2019 Medical Expenditure Panel Survey (MEPS) data. Payer analyses are only for trazodone prescriptions with diagnoses.

When comparing payer types among on-label and off-label prescriptions, significant differences emerge. Uninsured and self-pay individuals accounted for nearly 33% of off-label trazodone prescriptions, but they constituted only 23.9% of on-label trazodone prescriptions. Similarly, private payers comprise nearly 16.8% of off-label trazodone prescriptions, but only comprise 4.3% of on-label trazodone prescriptions. In contrast to the privately insured and self-pay figures, prescriptions reimbursed by public payers are more prevalent among on-label trazodone prescriptions compared with off-label prescriptions (71.6% vs 47.7%).

### Trazodone health spending

Table 3 presents total health care spending, total health plan spending, and total OOP spending on trazodone prescriptions overall and by on-label and off-label prescription status. Total health care spending is simply the sum of total health plan spending and total OOP spending. Table 3 shows that, in 2019, health care spending on trazodone in 2019 totaled nearly $294 million. Approximately 83.9% ($246.8 million) of this spending was attributed to off-label indications, while the remaining 16.1% ($47.2 million) was for on-label indications and trazodone prescriptions with no attached diagnoses. With respect to off-label spending, nearly 56% of total off-label spending is due to payer reimbursement, while 44% is due to OOP spending. The substantial OOP spending is attributable to uninsured/self-pay prescriptions, as uninsured/self-pay prescriptions comprise nearly 33.1% of all off-label trazadone prescriptions. Using the payer information we also calculated that Medicaid payor spending comprises approximately 21.7% of total spending ($53.5 million) on trazodone prescriptions. One limitation of these spending estimates is that, unlike Medicare and private payers, Medicaid receives manufacturer rebates for generic drugs.^32^ Combining calculations from the MEPS data with information from published sources^33^ we estimate that Medicaid rebates for trazodone amount to approximately $3.4 million, a small portion of the total spending on trazodone. We describe our calculations regarding the Medicaid rebate in greater detail in the Appendix.

**Table 3.** Trazodone health care spending off-label and on-label.

	Total spending (millions $)	Health plan (millions $)	Out-of-pocket (millions $)
Off-label diagnoses	246.8 [203.8–289.8]	138.3 [108.5–168.1]	108.5 [86.5–130.5]
On-label diagnoses	26.7 [16.2–37.3]	18.9 [10.2–27.6]	7.9 [4.0–11.7]
No diagnoses	20.5 [4.6–36.3]	15.0 [1.6–28.3]	5.5 [1.1–9.9]
Total	294 [244.9–343.1]	172.2 [137.5–206.8]	121.9 [98.1–145.6]

Source: 2019 Medical Expenditure Panel Survey (MEPS) data. 95% CIs in parentheses.

Finally, we examined per-prescription payer reimbursement for on-label vs off-label trazodone prescriptions with a simple comparison of means, followed by an adjustment for payer type and medication strength using regression analysis. In the simple comparison of means, we observed that pharmacies are typically reimbursed $10.66 for each off-label trazodone prescription. On the other hand, on-label trazodone prescriptions are reimbursed at a slightly lower rate of $9.64 per prescription, but this difference is not statistically significant. This calculation may be biased by variation in payer types and medication strengths across on-label and off-label trazodone prescriptions. To account for these differences, we performed ordinary least squares regression that adjusts for payer type and strength of medication. We found that adjustment for payer type and strength did not change the finding from the comparison of raw means. Although on-label trazodone prescriptions are estimated to be reimbursed $1.95 less than off-label trazodone prescriptions, this difference also failed to reach statistical significance at the 5% or 10% levels.

## Discussion

The results in this article indicate that approximately $247 million were spent on off-label use of trazodone; a decomposition of this estimate finds that nearly $195 million was spent on treatment of insomnia with trazodone in 2019. Randomized controlled trials for FDA drug approval typically cost $19 million^34^; thus, society, in just 1 year has spent approximately 10 times more on a drug than it would have cost to evaluate the effectiveness of the drug, suggesting a clearly inefficient equilibrium. Trazodone has been a treatment for insomnia for much of the previous decade. Even if only half of the 2019 amount was spent yearly over the 2010-2018 period, the implication is that society over the 2010-2019 period has spent nearly $1 billion on trazodone for insomnia—again, suggesting a clearly inefficient equilibrium. The $195 million spending estimate also illustrates the potential scale of the opportunity costs in the health care system, particularly if these expenditures do not yield clinical benefits.

Reasons for this equilibrium, where the effectiveness of trazodone for insomnia is uncertain, yet society spends hundreds of millions of dollars on its use, are 2-fold. First, pharmaceutical firms which are responsible for generating high-quality, RCT evidence to support FDA approval do not have a similar role for evidence generation in cases of off-label use. In such instances, the cost–benefit for firms to generate additional evidence for a new indication is potentially negative.^35,36^ In an extreme case, the results might show that trazodone is not effective with respect to insomnia, in which case firms will lose a significant revenue stream. In a neutral or positive case, a trial might show that trazodone is effective with respect to sleep and increases demand by a moderate amount. Even in this latter case the cost–benefit may skew negative since trazodone is already generic, and an increase in consumer demand might, in turn, induce additional firm entry. Second, in the off-label use case, because there is a need for evidence to resolve uncertainty regarding effectiveness, clinician investigators have taken the lead with respect to evidence generation. But, in many instances, these studies are likely not adequately funded and thus not appropriately powered.^24,25^ Instead of resolving uncertainty regarding effectiveness, results from these studies simply introduce more confusion regarding effectiveness of off-label treatments.^37^

A relevant question regarding our analysis is whether trazodone is representative of the broader set of drugs used off-label. While this is difficult to determine because few recent studies have examined off-label utilization and spending at the drug-indication level, there is reason to believe trazodone is not a unique case. For example, gabapentin is another generic drug that is primarily used for off-label indications.^38^ Without conducting a strict decomposition with respect to off-label use, we found that, in the 2019 MEPS data, excluding Medicaid payments, the United States spent nearly $2 billion on generic gabapentin. Concern regarding off-label uses of gabapentin has been present for more than 15 years. Gabapentin is another generic drug on which drug companies are unlikely to conduct trials with respect to non–FDA-approved indications due to a likely negative expected value from conducting the trial. Furthermore, existing research on gabapentin is also full of small sample-size studies, which introduce confusion with respect to the effectiveness of gabapentin. With respect to data that are available, Berger and colleagues,^2^ using data on drugs approved between 1995 and 2019, reported that off-label indications account for 43% of prescriptions within two-quarters of a drug's initial FDA approval. Similarly, data from Alexander et al,^3^ on off-label utilization and expenditures of atypical antipsychotics from 2008, suggest that trazodone is not an outlier with respect to aggregate spending and percentage of off-label use. Based on our review of the existing data, trazodone is likely to be an outlier with respect to total off-label prescriptions. This is because trazodone is both a highly utilized drug and, as we have shown, has a high proportion of off-label use.

## Policy solutions

Broadly, 2 significant problems lie at the heart of this inefficient state of off-label drug utilization. The first problem, as discussed, is that there is little to no high-quality clinical evidence supporting the effectiveness of trazodone on sleep health outcomes, and it is unlikely that such evidence will ever be produced. Similarly, in the unlikely case that evidence from an RCT were available in the near future, there remains a question of whether such evidence should have been available sooner. For example, a simple metric might be that society should expect evidence regarding off-label effectiveness be available as soon as spending on off-label use exceeds the cost of a credible trial. In the case of trazodone for sleep, such a threshold would have been crossed several years prior to 2019. A second problem is that society continues to spend nearly $247 million on trazodone for off-label conditions despite the lack of credible evidence to support that practice. Within this framework we consider new^39-42^ policy solutions broadly applicable to cases of off-label utilization of uncertain value. We focus on new solutions, which take advantage of advances in policy and modern data sources, but note that our suggestions are aimed at renewing discussion around a more efficient system of off-label use.

The first problem requires new methods to incentivize and expedite evidence generation. In the current environment, firms have mixed incentives to generate effectiveness information for off-label indications^35^; clinician researchers do not always have the funding (or possibly the interest) to conduct the necessary trials to generate information regarding off-label use. One potential solution to this problem is for agencies within the National Institutes of Health (NIH) to fund research aimed at evaluating outcomes for off-label utilization. Such a precedent exists, as an RCT funded by the NIH was instrumental in evaluating health outcomes related to the off-label use of Avastin in the treatment of macular degeneration.^43^ Publicly funded trials would also minimize the risk of potential price increases from industry-conducted trials.

Similarly, the Patient-Centered Outcomes Research Institute's (PCORI’s) statutory mission includes supporting research that compares the clinical effectiveness of interventions in real-world settings, with particular emphasis on areas characterized by high utilization and low evidentiary support—precisely the case with the widespread off-label use of trazodone for insomnia. Thus, this evidence gap is precisely the type of inefficiency that PCORI was designed to address by generating robust real-world evidence that is valid, relevant, and generalizable for clinical and policy decision-making. Considering this, we suggest that PCORI and similar agencies consider revisiting their funding eligibility criteria to allow for greater flexibility in supporting comparative effectiveness research on high-priority off-label uses, particularly when such uses may represent de facto standards of care.

Finally, given the significant health plan spending on trazodone for the treatment of insomnia, it would also be in the interest of insurers to conduct an RCT aimed at evaluating the effectiveness of this treatment.^44^ As a historical example, Insurers previously provided funding to evaluate the effects of autologous stem cell transplantation as a treatment for breast cancer.^45^ In the case that trazodone is effective for insomnia, the implication is that current levels of utilization are justified. Alternatively, finding that trazodone is ineffective for treating insomnia would imply that even a 17% reduction in trazodone usage (in just 1 year) would justify health plan spending on the trial.

A second potential solution relates to pharmaceutical pricing and addresses the issue of health spending of uncertain value. Since FDA approval of novel pharmaceuticals relies on efficacy and safety data and drug launch prices are dependent on a given product's efficacy with respect to its initial approved indication,^46^ prices for off-label indications of uncertain value should be lower than the price for the on-label indication. Medicare currently negotiates price with manufacturers for a limited number of drugs. Medicare should factor in off-label use of uncertain value in their pricing negotiations. While Medicare is currently limited with respect to the drugs that it can negotiate, private payers have no such restrictions and can immediately negotiate drug prices based on the level of off-label utilization—in particular, the level of off-label utilization of uncertain (or no) value. While MEPS data are, in many ways, ideal for calculating off-label use, MEPS sample sizes of approximately 30 000 individuals means that other data sources will likely be necessary to capture off-label use in low-incidence conditions (eg, cancer). In these cases, off-label utilization can be calculated from modern data sources such as electronic health record data. This second approach, aimed at lowering drug prices, while unlikely to lead directly to lower off-label utilization, offers the immediate benefit of lowering health care spending for off-label use.

A third potential solution relates to reimbursement. The trazodone reimbursement results show that health plan reimbursement to pharmacies is nearly identical for on-label and off-label indications. Since off-label utilization in cases such as trazodone has no proven value, off-label drugs without any proven benefit should be reimbursed at a lower rate. With respect to generics, health plans can lower the “ingredient” rate, which is dependent on the prices that pharmacies pay to distributors and manufacturers. A lower “ingredient” rate might also make pharmacies negotiate lower prices with pharmaceutical companies for drugs with high rates of off-label use. Physicians do not benefit directly from prescribing trazodone but do benefit from prescribing and treatment behavior with respect to office-based therapies such as chemotherapies. This approach offers the benefit of lowering health care spending and, in cases of physician-administered drugs, may also lead to decreased near- and long-term utilization by providers. A drawback of this proposed solution is that lowering reimbursement for generics, which are already priced close to the marginal cost of production, has the potential to negatively impact pharmacy profits and viability.

## Conclusion

Off-label utilization, especially in cases of uncertain or low-value care, has the potential to increase wasteful societal health care spending and negatively impact patient outcomes. In 2019, health care spending on off-label uses of trazodone was nearly $247 million. Off-label use of trazodone for sleep disorders accounted for nearly $195 million of that spending, despite the fact that trazodone's effectiveness on sleep is unknown. A high-quality randomized trial to measure the effectiveness of trazodone on sleep can be conducted for significantly less than total spending on trazodone in just 1 year. We observed early evidence that this is not an uncommon occurrence in the off-label space. We proposed 3 potential policy solutions that are implementable with modern data sources and aimed at a more efficient system of off-label use.

## Supplementary Material

qxaf114_Supplementary_Data

## Data Availability

MEPS data is publicly available data and can be downloaded from the Agency for Healthcare Research and Quality website.
