# Supplementary material for: Off-label policy through the lens of trazodone usage and spending in the United States
Source: Health Aff Sch. 2025 Jun 11;3(7):qxaf114. doi: 10.1093/haschl/qxaf114 (PMC12278056; doi:10.1093/haschl/qxaf114)
Supplement: qxaf114_Supplementary_Data [file qxaf114_supplementary_data.zip › Appendix_Health_Affairs_Scholar.docx]

**Appendix**

One issue with the Trazodone spending estimates is that, unlike Medicare and private payers, Medicaid receives manufacturer rebates for generic drugs. Thus, a part of the payer expenditure data captured by the MEPS data, is returned to Medicaid.

In 2019, the Medicaid generic drugs rebate amount was fixed at 13% of the Average Manufacturers Price (AMP), plus a possible inflationary rebate. To identify the AMP we used information from the 2019 National Average Drug Acquisition Cost (NADAC) files, which reports the average price paid by pharmacies to manufacturers for generic drugs. We assigned NADAC prices to all claims reimbursed by Medicaid in the MEPS data and then calculated a 13% rebate for all Medicaid claims. This flat rebate reduction amounts to a total savings of approximately $1.67 million, which compared to the $53.5 million Medicaid spent on trazodone. In addition to the statutory rebates, Medicaid may also receive an inflationary rebate and supplemental rebates on top of the statutory rebate. In 2019 the Medicaid and CHIP Payment and Access Commission (MACPAC) calculated that inflationary rebates for generics were equivalent to the statutory rebate. MACPAC Supplemental rebates were calculated to be approximately 3.7% of the statutory rebate, approximately another $62,000. Thus, Medicaid rebates for trazodone (~$3.4 million) have little material effect on our initial conclusions regarding on-label and off-label spending.

**Citations**

Dolan, R. (2019). Understanding the Medicaid Prescription Drug Rebate Program. Kaiser Family Foundation.<https://www.kff.org/wp-content/uploads/2019/11/Understanding-the-Medicaid-Prescription-Drug-Rebate-Program-updated-3.2021.pdf>

Park C., Trends in Medicaid Drug Spending and Rebates. MACPAC. October 27, 2022
